# Highly Efficient and Reusable Copper-Catalyzed *N*-Arylation of Nitrogen-Containing Heterocycles with Aryl Halides

**DOI:** 10.3390/molecules14125169

**Published:** 2009-12-10

**Authors:** A Young Kim, Hyun Ju Lee, Ji Chan Park, Hyunju Kang, Haesik Yang, Hyunjoon Song, Kang Hyun Park

**Affiliations:** 1 Department of Chemistry and Chemistry Institute for Functional Materials, Pusan National University, Busan 609-735, Korea; E-Mails: cuteangel02@hanmail.net (AY.K.); hjloveth486@naver.com (H.J.L.); asteria818@hanmail.net (H.K.); hyang@pusan.ac.kr (H.Y.); 2 Department of Chemistry, Korea Advanced Institute of Science and Technology, Daejeon, Korea; E-Mails: whiteprince@kaist.ac.kr (J.C.P.); hsong@kaist.ac.kr (H.S.)

**Keywords:** *N*-arylation, copper, heterocycles, heterogeneous catalyst, acetylene black

## Abstract

CuO/AB was found to be a simple and efficient catalyst for the *N*-arylation of a variety of nitrogen-containing heterocycles, giving the products in excellent yields.

## 1. Introduction

**Scheme 1:**
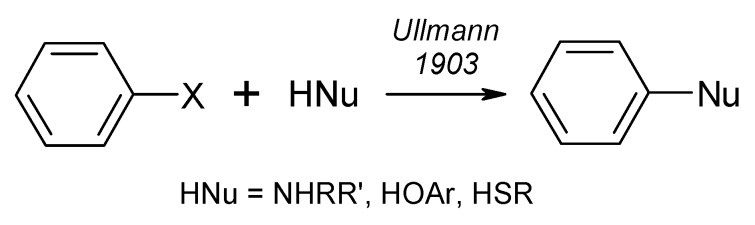
Ullmann condensation.

The copper-mediated C-heteroatom (C-N, C-O, C-S, C-P, C-Se), C-C, and C-metal bonds formations are pivotal transformations that have been developed to include a wide range of substrates. Specifically, the *N*-arylation of nitrogen-containing heterocycles is of particular interest as the resulting products represent important structural motifs of numerous natural products and biologically active compounds. *N*-Arylimidazoles, *N*-arylpyrroles, *N*-arylpyrazoles, *N*-arylindoles, and *N*-aryl-triazoles have received substantial attention in a variety of fields throughout the chemical, pharmaceutical, and material sciences [1,2,3,4,5,6]. Despite the significant progress made in the development of copper catalyzed coupling reactions of this type, there still exists a need for new methods that involve cheap and `environmentally sound catalysts [7,8,9]. Classical Ullmann chemistry, along with closely related methods, have been known for a full century. This copper-mediated synthesis of biaryl is known as the *Ullmann condensation* (synthesis of diaryl ethers, diaryl amines, or diaryl thioethers). (Scheme 1). During the first 70 years of the 20^th^ century, copper was nearly the only metal usable for aryl-aryl bond formation, finding initial in the reductive symmetrical coupling of aryl halides, corresponding aromatic compounds, aryl halides, diaryl ether, *N*-containing reactants, phenols, and related nucleophilic agents [10,11,12,13,14,15,16,17,18]. Relatively mild and highly efficient CuI-catalyzed *N*-arylation procedures for nitrogen-containing heterocycles (imidazoles, benzimidazoles, pyrroles, pyrazoles, indoles, triazoles) with aryl and heteroaryl halides have also been developed [19,20,21,22]. In this paper, the CuO hollow nanospheres were used to catalyze the *N*-arylation of nitrogen-containing heterocycles with aryl halides. 

## 2. Results and Discussion

In the present study, an approach for gram-scale synthesis of uniform Cu_2_O nanocubes by a one-pot polyol process was developed [23]. The CuO hollow nanospheres were prepared by adding an aqueous ammonia solution to the Cu_2_O nanocube colloidal solution. The CuO hollow nanospheres were then immobilized onto acetylene black (AB) or charcoal. Thus, these immobilized CuO hollow nanospheres overcome the issue of reuse [24].

### 2.1. Catalyst characterization

**Figure 1:**
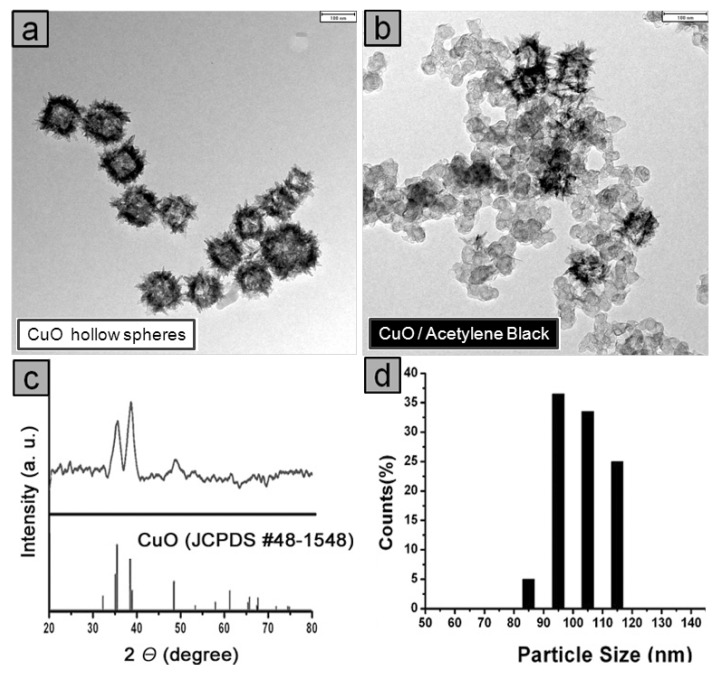
(a) TEM image, c) XRD pattern, d) size distribution diagrams of the CuO hollow nanospheres, and (b) TEM image of the CuO hollow nanospheres on acetylene black (CuO/AB). The scale bars represent 100 nm.

The Cu_2_O nanocubes prepared by a polyol process were transformed into CuO hollow nanospheres by a controlled oxidation reaction using an aqueous ammonia solution. Addition of ammonia solution (2.0 mL, 3.7 M) into a Cu_2_O colloidal solution gave the CuO hollow nanospheres. The transmission electron microscopy (TEM) image in Figure 1a shows the regular hollow shape of the CuO particles. The CuO hollow spheres were obtained as (103 ± 8)-nm-sized, highly monodisperse nanoparticles (Figure 1d). The crystalline features of the hollow spheres are represented in the XRD data (Figure 1c). The main peaks at *θ* = 35^o^ and 39^o^ were assigned to the reflections of the (002)/(11–1) and (111)/(200) planes in the CuO phase (JCPDS No. 48-1548). The CuO hollow particles were immobilized on acetylene carbon black through a simple sonication method at room temperature. The TEM image in Figure 1b shows that the immobilized CuO hollow spheres were well dispersed, isolated, and maintained their original size and structure with an average diameter of approximately 100 nm. The absolute amount of copper metal on the acetylene black was determined by inductively coupled plasma-atomic emission spectroscopy (ICP-AES). The CuO hollow spheres on the acetylene black showed excellent activity towards a wide range of azides and acetylenes.

### 2.2. Reaction test

**Scheme 2:**
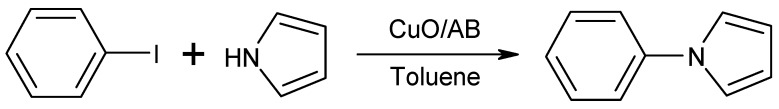
Test reaction.

Prior research studies that have tested catalyst effectiveness have employed iodobenzene and pyrrole as benchmark substrates (Scheme 2).

The *N*-arylation of iodobenzene (0.17 mL, 1.5 mmol) and pyrrole (0.15 mL, 2.25 mmol) with CuO/AB in toluene (7.0 mL), afforded 1-phenyl-*1H*-pyrrole. As shown in Table 1, 3.0 mol% CuO/AB in toluene at 180 °C for 8 h gave 1-phenyl-*1H*-pyrrole in 51% yield (entry 1, Table 1). When the reaction time was increased to 18 h, an 81% conversion was achieved (entry 2, Table 1). The reaction temperature was increased to 180 °C through use of a stainless steel reactor. Furthermore, when 5.0 mol% of the catalyst was used, a 22% yield was achieved at 150 °C for, 8 h (entry 3, Table 1). In general, it was found that increasing reaction temperature and time were effective means of increasing the conversion (59% for 180 °C, 8 h, entry 4, Table 1). Increasing the reaction time and changing the base to KOH, K_2_CO_3_, and KO*^t^*Bu, gave respective, varied yields of 9, 71 and 72% (entries 5, 6, and 7, Table 1). After that, in order to exploit the thermally stable properties of the CuO/AB catalyst, reaction temperature was increased. Placing the reaction in a closed system (stainless steel reactor) at 180 °C and reacting for 18 h achieved a 92% conversion (entry 8, Table 1). Finally, the optimum reaction conditions were achieved: *N*-arylation of iodobenzene (0.17 mL, 1.5 mmol) and pyrrole (0.15 mL, 2.25 mmol) with CuO/AB (70 mg, 5.0 mol%) in Toluene (7.0 mL) at 180 °C for 18 h to afford 1-phenyl-1*H*-pyrrole. In addition, under optimum reaction conditions, no reaction occurred without a catalyst. It was also observed via NMR, TLC and column chromatography that no side products, such as Ar-Ar coupling products, were formed. Remarkably, after the reaction, the CuO on AB as separated by centrifugation and could be reused ten times under the same reaction conditions, without any loss of catalytic activity; the maximum reusability has not yet been tested. These results confirm that the catalytic system presented herein satisfies the conditions for heterogeneous catalysts of ease of separation, recyclability, and persistence. 

**Figure 2:**
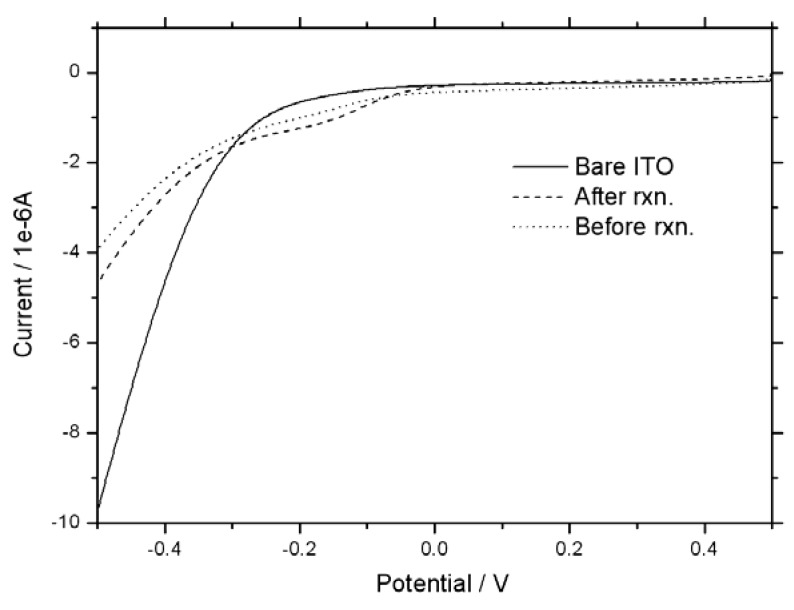
Cyclic voltammograms (CVs) of CuO hollow nanospheres on acetylene black, before (Dot line) and after the two cycle. (Dash line).

As transmission electron microscope (TEM) and cyclic voltammograms (CVs) studies prove, the structure of the CuO hollow nanospheres on acetylene black (CuO/AB) remained unchanged after the reaction, which is demonstrating catalyst recyclability (Figure 2). CV were measured by using CHI 405A (CH Instruments). 70 μL of a CuO in ethanol was dropped to ITO electrode. After 3 h, the signal of the reduced CuO on ITO was measured via linear sweep voltammetry. The linear sweep was voltamogram obtained at CuO ITO electrode in 10 mM tris buffer (pH 7.4) at scan rate of 50 mV/s.

Furthermore, in the case of using various nitrogencontaining heterocycles, good results were achieved (Table 2). A wide variety of substrates with C-I, C-Br, and C-Cl bonds, as well as nucleophiles, have been examined. Relative reactivities of halogen-substituted substrates toward the halophilic attack by a carboanion were also investigated. The following order of relative reactivities toward the halophilic attack was ascertained: R-I, R-Br or R-Cl, with a bond reactivity order of: C-I > C-Br > C-Cl (entries 1~3, Table 2). This new catalytic system was also suitable for other electron-rich nitrogen heterocycles. Experiments on substituted aryl halides were conducted with OH-, OMe-, and CH_3_-containing substrates. The results were showing 70% conversion yield in case of 4-bromo-phenol while only a low yield was obtained in case of 1-bromo-4-methoxybenzene. In one case, the use of 1-chloro-4-methylbenzene resulted in lower obtained yields (entry 6, Table 2). Unfortunately, no reaction occurred in case of *p*-chloroacetophenone. It is worth noting that these optimized conditions could be applied to the *N*-arylation of other imidazole derivatives. (pyrroles, pyrazoles, imidazoles; Table 2). For example, the *N*-arylation of 1*H*-pyrazole with iodobenzene afforded the corresponding 1-phenyl-*1H*-pyrazole in 96% conversion yield (entry 7, Table 2). In addition, the coupling of 1*H*-imidazole with iodobenzene affords the 1-phenyl-1*H*-imidazole. Further investigation of the reaction of iodobenzene with other heteroarylamines, as well as aniline, under the optimized conditions was also undertaken. Pyridin-3-amine, and pyrimidin-2-amine heteroarylamines gave the expected diphenylamine, *N*-phenylpyridin-3-amine, and *N*-phenylpyrimidin-2-amine with good conversion (entries 11 and 12, Table 2). Such a result shows a similar yield albeit with shorter reaction time (24 h–40 h), compared with known literature [25,26,27,28].

## 3. Experimental

### 3.1. General remarks

Reagents were purchased from Aldrich Chemical Co. and Strem Chemical Co. and used as received. Reaction products were analyzed by ^1^H-NMR. ^1^H-NMR spectroscopy obtained on a Varian Mercury Plus (300 MHz). Chemical shift values were recorded as parts per million relative to a tetramethylsilane internal standard, unless otherwise indicated, and coupling constants in Hertz. Reaction products were assigned by comparison with the literature value of known compounds. The CuO and CuO nanoparticles immobilized on acetylene black were characterized by TEM (Philips F20 Tecnai operated at 200 kV, KAIST). Samples were prepared by placing a few drops of the corresponding colloidal solution on carbon-coated copper grids (Ted Pellar, Inc.). The X-ray powder diffraction (XRD) patterns were recorded on a Rigaku D/MAX-RB (12 kW) diffractometer. The copper loading amounts were measured by inductively coupled plasma-atomic emission spectrometry (ICP-AES).

### 3.2. General procedure for catalytic N-arylation of nitrogen-containing heterocycles with aryl halides

In a 25 mL stainless steel reactor, CuO hollow nanospheres on acetylene black (CuO/AB) (70 mg, 5.0 mol% with respect to the substrate concentration), iodobenzene (0.17 mL, 1.5 mmol), pyrrole (0.15 mL, 2.25 mmol), KO*^t^*Bu (0.34 g, 3.0 mmol), and toluene (7.0 mL) were added. The mixture was stirred for 18 h at 180 °C. After the reaction, the nanoparticles were separated from the clean solution by centrifugation and the clean solution analyzed by 300 MHz NMR.

### 3.3. Synthesis of CuO hollow nanospheres

The CuO hollow nanospheres were synthesized by a controlled oxidation reaction of Cu_2_O nanocubes. Typically, Cu_2_O nanocubes were prepared by a polyol process in 1,5-pentanediol (PD, Aldrich, 96%) in the presence of poly(vinyl pyrrolidone) (PVP, Aldrich, M_w_ = 55,000). The PVP (5.3 g) dissolved in 45.0 mL of 1,5-pentanediol (PD, Aldrich, 96%), was slowly heated to 240 °C under a nitrogen atmosphere. Then, 4.0 mmol of Cu(acac)_2_ (Strem, 98%), dissolved in 15 mL of PD, was injected into the hot PVP solution at 240 °C and the mixture allowed to stir for 15 min at the same temperature. The yellowish colloidal dispersion was cooled to room temperature and precipitated by adding acetone followed by centrifugation at 8,000 rpm for 20 min. The precipitated Cu_2_O particles were washed with ethanol several times and re-dispersed in ethanol. To obtain the CuO hollow nanospheres, an aqueous ammonia solution (2.0 mL, 3.7 M) was added into 25.0 mL of the Cu_2_O cube dispersion in ethanol (16.0 mM with respect to the precursor concentration). The mixture was then stirred at room temperature for 2 h. After the reaction, the final products were collected by centrifugation at 6,000 rpm for 20 min.

### 3.4. Immobilization of CuO hollow nanospheres on acetylene carbon black (CuO/AB) and charcoal (CuO/C)

The acetylene carbon black (Strem, 99.99%, 1.2 g) was mixed with the CuO hollow nanosphere dispersion in ethanol (100 mL, 17.0 mM), and the reaction mixture sonicated for 1 h at room temperature. After 1 h, the product CuO/AB was washed with ethanol several times and vacuum dried at room temperature. For the synthesis of CuO/C, the mixture solution of charcoal (0.8 g) and CuO hollow nanosphere dispersion in ethanol (50.0 mL, 50.0 mM) was refluxed for 4 h. After 4 h, the black suspension was cooled to room temperature and precipitated by centrifugation. The product CuO/C was washed with ethanol thoroughly and dried in a vacuum oven at room temperature.

## 4. Conclusions

In conclusion, an environmentally sound process for CuO/AB catalyzed *N*-arylation has been developed, for a variety of nitrogen-containing heterocycles with aryl halides. In addition, the CuO/AB was readily separated by centrifugation and could be reused ten times under the present reaction conditions without any loss of catalytic activity. Transition metals loaded on acetylene black were useful reagents for a wide variety of organic transformations. Moreover, these heterogeneous systems are promising industrial catalysts with the concept of chemical economy. Further studies to expand the applications of the catalytic system are underway in this laboratory.

## Figures and Tables

**Table 1:** Screening reaction condition for *N*-arylation of iodobenzene with pyrrole.

Entry	Cat (mol %)	Temp (°C)	Time (h)	Base	Conv. (%)^a^
1	3 mol%	180	8	KO*^t^*Bu	51
2	3 mol%	180	18	KO*^t^*Bu	81
3	5 mol%	150	8	KO*^t^*Bu	22
4	5 mol%	180	8	KO*^t^*Bu	59
5	5 mol%	180	12	K_2_CO_3_	9
6	5 mol%	180	12	KOH	71
7	5 mol%	180	12	KO*^t^*Bu	72
8	5 mol%	180	18	KO*^t^*Bu	96
9	recovered from # 8	180	18	KO*^t^*Bu	94
10	recovered from # 9	180	18	KO*^t^*Bu	92
11	recovered from # 10	180	18	KO*^t^*Bu	92
12	recovered from # 11	180	18	KO*^t^*Bu	90
13	recovered from # 12	180	18	KO*^t^*Bu	96
14	recovered from # 13	180	18	KO*^t^*Bu	96
15	recovered from # 14	180	18	KO*^t^*Bu	94
16	recovered from # 15	180	18	KO*^t^*Bu	96
17	recovered from # 16	180	18	KO*^t^*Bu	87

^a^ Determined by ^1^H-NMR. Yields are based on the amount of iodobenzene used.

**Table 2:**
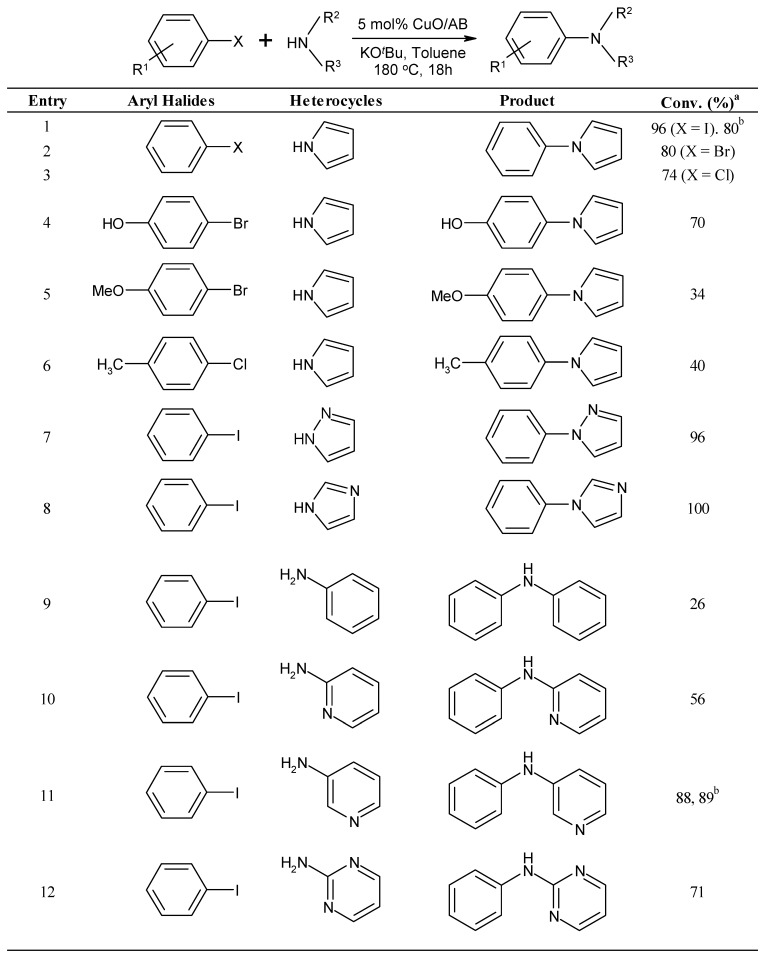
CuO/AB catalyzed *N*-arylation of various Nitrogen-containing Heterocycles with Aryl Halides.

^a^ Determined by ^1^H-NMR. Yields are based on the amount of aryl halides used; ^b^ Yields are isolated yields after column chromatography on silica.
